# Patient characteristics and patient flow in a small accident and emergency department

**DOI:** 10.1186/1757-7241-23-S1-A37

**Published:** 2015-07-16

**Authors:** Mohamad Jawhara, Niels-Henrik Staalsen

**Affiliations:** 1 Surgical Department, Thy-Mors Hospital, Thisted, Denmark; 2 Emergency Department, Thy-Mors Hospital, Thisted, Denmark

## Background

Only few data exist on patient characteristics and flow within Acute & Emergency departments (A&E) in Denmark. Neither do we have solid data on the effectivity, i.e. how many patients are readmitted with the same diagnosis within a short time.

## Aim

To provide data on patient characteristics and patient flow within our A&E department.

## Methods

The study period was from February 1st until May 30th 2014. All patients admitted to our department (3,006) were included. Orthopedic patients with smaller wounds and fractures were excluded. Patient characteristics (gender, age, primary diagnosis, date, and length of hospital stay, medical specialty, follow-up, and re-admittance within 30 days) were registered.

## Results

60% were admitted to the Medical Department, 22% to the Surgical Department, 15% to the Orthopedic Department and 3% to Department of Gynecology. 51% were women and 49% were men. Medical and Orthopedic patients were typically above 51 years of age while gynecological patients typically were between 21 and 40 years old. The surgical patients had an equal age distribution within the interval 21-90 years. No week-week variations of admittance (mean 25 patients a day) were seen. 59% of the patients were treated within 48 hours in the A&E department - out of these 59%, 25% were discharged immediately, 35% were send to the clinical departments, the rest were sent to another hospital or died. Out of patients treated in the A&E department, 60% (Surgery), 39% (Medicine), 41% (Orthopedic), and 7 8% (Gynecology) were discharged without need of follow-up by General Practitioner (GP). 16% (Surgery), 10% (Medicine), 20% (Orthopedic Surgery), and 30% (Gynaecology) were seen in the outpatient clinics. 5.5% (Surgery), 1.9% (Medicine), 5.7% (Orthopedic Surgery), and 7.9 (Gynecology) were sent to the outpatients clinic of a different specialty. Totally, 4.3% were readmitted within 30 days once and 0.7% twice.

## Conclusions

In this descriptive study we have provided data on patient characteristics and patient flow in our A&E department during a 4 month study period.

